# Postoperative Remote Automated Monitoring and Virtual Hospital-to-Home Care System Following Cardiac and Major Vascular Surgery: User Testing Study

**DOI:** 10.2196/15548

**Published:** 2020-03-18

**Authors:** Michael McGillion, Carley Ouellette, Amber Good, Marissa Bird, Shaunattonie Henry, Wendy Clyne, Andrew Turner, Paul Ritvo, Sarah Ritvo, Nazari Dvirnik, Andre Lamy, Richard Whitlock, Christopher Lawton, Jake Walsh, Ken Paterson, Janine Duquette, Karla Sanchez Medeiros, Fadi Elias, Ted Scott, Joseph Mills, Deborah Harrington, Mark Field, Prathiba Harsha, Stephen Yang, Elizabeth Peter, Sanjeev Bhavnani, PJ Devereaux

**Affiliations:** 1 School of Nursing McMaster University Hamilton, ON Canada; 2 Population Health Research Institute Hamilton, ON Canada; 3 McMaster University Hamilton, ON Canada; 4 Hope for the Community CIC Coventry United Kingdom; 5 Coventry University Coventry United Kingdom; 6 York University Toronto, ON Canada; 7 Department of Surgery McMaster University Hamilton, ON Canada; 8 Hamilton Health Sciences Hamilton, ON Canada; 9 Cardiac and Vascular Program Hamilton Health Sciences Hamilton, ON Canada; 10 Liverpool Heart and Chest Hospital Liverpool United Kingdom; 11 McGill University Montreal, ON Canada; 12 Lawrence S. Bloomberg Faculty of Nursing University of Toronto Toronto, ON Canada; 13 Scripps Clinic & Research Foundation San Diego, CA United States; 14 Departments of Health Research Methods, Evidence, and Impact (HEI) and Medicine McMaster University Hamilton, ON Canada

**Keywords:** monitoring, physiologic, postoperative care, user testing

## Abstract

**Background:**

Cardiac and major vascular surgeries are common surgical procedures associated with high rates of postsurgical complications and related hospital readmission. In-hospital remote automated monitoring (RAM) and virtual hospital-to-home patient care systems have major potential to improve patient outcomes following cardiac and major vascular surgery. However, the science of deploying and evaluating these systems is complex and subject to risk of implementation failure.

**Objective:**

As a precursor to a randomized controlled trial (RCT), this user testing study aimed to examine user performance and acceptance of a RAM and virtual hospital-to-home care intervention, using Philip’s Guardian and Electronic Transition to Ambulatory Care (eTrAC) technologies, respectively.

**Methods:**

Nurses and patients participated in systems training and individual case-based user testing at two participating sites in Canada and the United Kingdom. Participants were video recorded and asked to think aloud while completing required user tasks and while being rated on user performance. Feedback was also solicited about the user experience, including user satisfaction and acceptance, through use of the Net Promoter Scale (NPS) survey and debrief interviews.

**Results:**

A total of 37 participants (26 nurses and 11 patients) completed user testing. The majority of nurse and patient participants were able to complete most required tasks independently, demonstrating comprehension and retention of required Guardian and eTrAC system workflows. Tasks which required additional prompting by the facilitator, for some, were related to the use of system features that enable continuous transmission of patient vital signs (eg, pairing wireless sensors to the patient) and assigning remote patient monitoring protocols. NPS scores by user group (nurses using Guardian: mean 8.8, SD 0.89; nurses using eTrAC: mean 7.7, SD 1.4; patients using eTrAC: mean 9.2, SD 0.75), overall NPS scores, and participant debrief interviews indicated nurse and patient satisfaction and acceptance of the Guardian and eTrAC systems. Both user groups stressed the need for additional opportunities to practice in order to become comfortable and proficient in the use of these systems.

**Conclusions:**

User testing indicated a high degree of user acceptance of Philips’ Guardian and eTrAC systems among nurses and patients. Key insights were provided that informed refinement of clinical workflow training and systems implementation. These results were used to optimize workflows before the launch of an international RCT of in-hospital RAM and virtual hospital-to-home care for patients undergoing cardiac and major vascular surgery.

## Introduction

### Background

Cardiac and major vascular surgeries are common surgical procedures associated with high rates of postsurgical complications and related hospital readmission [1,2]. A North American prospective cohort study (involving 5158 patients) by the National Institutes of Health and Canadian Institutes of Health Research Cardiothoracic Surgical Trials Network found that 18.7% of cardiac surgery patients were readmitted within 60 days [3]. The most common drivers of first readmission included infection, arrhythmia, and fluid volume overload [3]. Data from the US 2014 registry (N=11,246) reported comparable rates of unplanned 30-day readmission among major vascular surgery patients, for example, infrainguinal bypass: 15.7% [4]. Recent retrospective data from Boston Medical Centre, reporting on patients (N=649) having a range of major vascular surgeries (eg, endovascular lower extremity procedures and carotid or cerebrovascular procedures), demonstrated that 21% of patients were readmitted within 30 days. Postsurgical complications accounted for 35.5% of these readmissions, driven most commonly by surgical site infections, graft failures, and bleeding [5].

A factor contributing to high postsurgical complications and readmission rates following cardiac and major vascular surgeries is inadequacy of current systems for patient monitoring in hospital and at home [6-10]. Routine nursing surveillance of patients on hospital surgical wards includes manual vital signs assessments every 4 to 12 hours [6,10]. On the basis of such infrequent vital signs measurements, extrapolations are made about the stability of patients’ physiologic status for extended periods of time [11]. As a result of these practices, the incidences of patient hemodynamic compromise and instability are often missed, as are opportunities to facilitate timely clinician response and early intervention [8-12]. In a study examining the incidence of postoperative hypotension, Turan et al [13] found that 18% of patients on surgical wards had an episode of mean arterial pressure <65 mm Hg for a minimum of 15 min. When taking routine, manual vital signs observations every 4 hours, nurses missed approximately half of all these episodes.

The problem is further compounded once patients are discharged home without surveillance or health professional support—a significant number of patients sustain complications that their surgical teams are unaware of. A prospective study (N=328) in the United Kingdom found that 28% of cardiac surgery patients required urgent physician or district nurse intervention within the first 6 weeks of recovery at home [6]. Of these patients, 21% required hospital readmission because of major complications including cardiac arrhythmia, pneumonia, renal failure, or sternal wound infections. Patients’ respective surgical care teams were unaware of any such complications requiring treatment [6].

Increasing efforts are being made to implement postoperative remote automated monitoring (RAM) and surveillance systems to improve patient outcomes through facilitation of continuous patient monitoring, early detection of deterioration, and remote patient support [9,11]. Sophisticated RAM and virtual care deployments include wireless sensors worn by the patient and supported by network infrastructure to acquire, transmit, and integrate continuous physiologic data [9,11]. Synthesis of this information is typically driven by the hospital early warning systems that direct action of frontline nursing staff, including escalation of care to the most responsible physician or rapid response team [9].

RAM surveillance systems have major potential, but the science of implementing and evaluating these systems is complex and still at an early stage [9]. Although a number of recent studies report that nurses and physicians support the need for RAM technologies on surgical wards and into the home setting, there are conflicting views which convey both excitement and apprehension about the consequences of such systems on clinical workflows and outcomes, as well as the experience of patient care [14-17]. In a study to solicit clinical staff perspectives of the introduction of RAM on general and surgical wards, Prgomet et al [14] found that while RAM technologies were viewed by nurses and physicians as potentially advantageous to the identification of early patient deterioration, a number of concerns were raised about possible drawbacks. Clinicians expressed worry that RAM technologies would decrease meaningful patient contact, reduce flexibility about the use of personal clinical judgement, and result in unwanted patient anxiety and discomfort [14]. Other studies have supported similar results, stressing the need for targeted training, educational opportunities, and pilot user testing studies to allow clinicians and patients to get familiar and comfortable with RAM systems and provide feedback [15,17].

### Objectives

Our team is conducting a randomized controlled trial (RCT) of a RAM and virtual hospital-to-home intervention entitled, TecHnology-Enabled remote monitoring and Self-MAnagemenT—VIsion for patient EmpoWerment following Cardiac and major VasculaR surgery (THE SMArTVIEW, CoVeRed) [9,18,19]. The SMArTVIEW intervention combines RAM and virtual hospital-to-home support using Philips monitoring technologies.

The current RCT (N=800) [1,9] will examine the impact of SMArTVIEW on patients aged 65 years and older at two hospital sites (Canada and the United Kingdom) on an array of clinical and feasibility outcomes, including a composite of 45-day hospital readmission and emergency department and urgent care center visits; postoperative complications; patient-reported outcomes; and intervention adherence [9,18,19].

Recognizing that implementation of RAM represents a change to typical postoperative care [9,14-17], the purpose of this study was to examine user performance and acceptance of RAM and virtual care technologies planned for use in the SMArTVIEW trial [9,18,19].

## Methods

### The TecHnology-Enabled Remote Monitoring and Self-MAnagemenT—VIsion for Patient EmpoWerment Remote Automated Monitoring and Virtual Care Intervention

This user testing study (and the subsequent SMArTVIEW trial) was in response to a call for applications to the Canadian Institutes of Health Research eHealth Innovation Partnerships program. This funding opportunity was designed to facilitate experimental, *real-world*, large-scale implementations, focusing on the integration of existing innovations that are beyond the prototyping stage and ready for deployment in the real-world conditions. To this end, we extended an invitation to vendors, through various hardware and software consortiums in North America, to showcase their market-ready solutions that could support our RAM and virtual care needs. Vendor choice was based on ability to contribute equipment and personnel time for training study staff, as well as the availability of products that were at a minimum *technology readiness level* 7 to 9 (0=early prototyping, 9=ready for use under operational conditions), according to Innovative Solutions Canada.

Our aim was to implement an end-to-end solution that incorporates both RAM in hospital and virtual hospital-to-home recovery support for the first 30 days at home, following cardiac and major vascular surgery. Although multiple vendors came forward with various solutions, Philips was in a position to provide market-ready, configurable technology solutions for both hospital RAM and hospital-to-home virtual care that could be packaged together in an *end-to-end* solution for immediate deployment.

**Figure 1:**
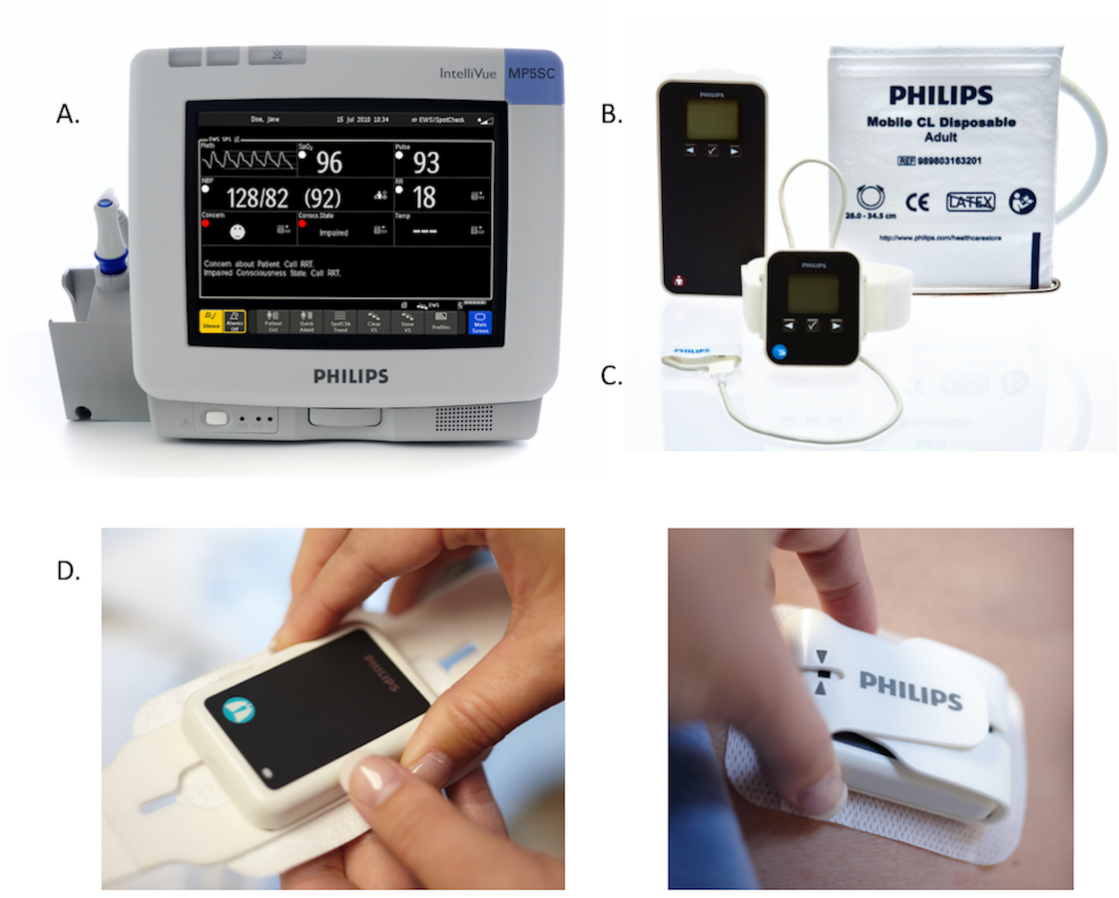
The Philips Guardian Solution. (A) MP5 spot-check monitor, (B) wireless blood pressure monitor, (C) wireless continuous pulse oximetry monitor, and (D) wireless respiratory sensor. Reproduced with permission from Philips Canada (Markham, ON) (reprinted with copyright permission from the publisher).

On the surgical ward, RAM is supported by the Philips Guardian solution as illustrated in Figure 1 [1,9,18]. Guardian includes a central trending monitor at the nursing station; a bedside, portable spot-check vital signs monitor (MP5); and three wireless wearable patient sensors, which communicate with the MP5 bedside monitor and the Guardian central monitor via short-range radio and Wi-Fi, respectively. A wireless sensor applied to the index finger and wrist monitors continuous blood oxygen saturation (SpO_2_) and pulse rate, an inflatable cuff module measures noninvasive blood pressure, and a small pod applied to the left costal arch measures respiration rate and patient position as shown in Figure 1. The Philips Guardian solution is programmable according to hospital early warning score parameters—vital signs data are integrated automatically to calculate the patient’s early warning score [1,9,18]. Hospital warning scores are used to identify patients with early signs of clinical deterioration to facilitate prompt intervention and prevent a major adverse event [9]. In the event that a patient’s early warning score triggers the need for prespecified clinical action, a notification is sent to the ward nurse via a handheld device (eg, Android phone), calling for early attention to care [1,9,18]. To safeguard against notification fatigue, the system features built-in trend analyses and reassurance measurements in order to verify signals that are indicative of early patient deterioration. For more information on hospital early warning systems, the readers are referred to McGillion et al [9].

**Figure 2:**
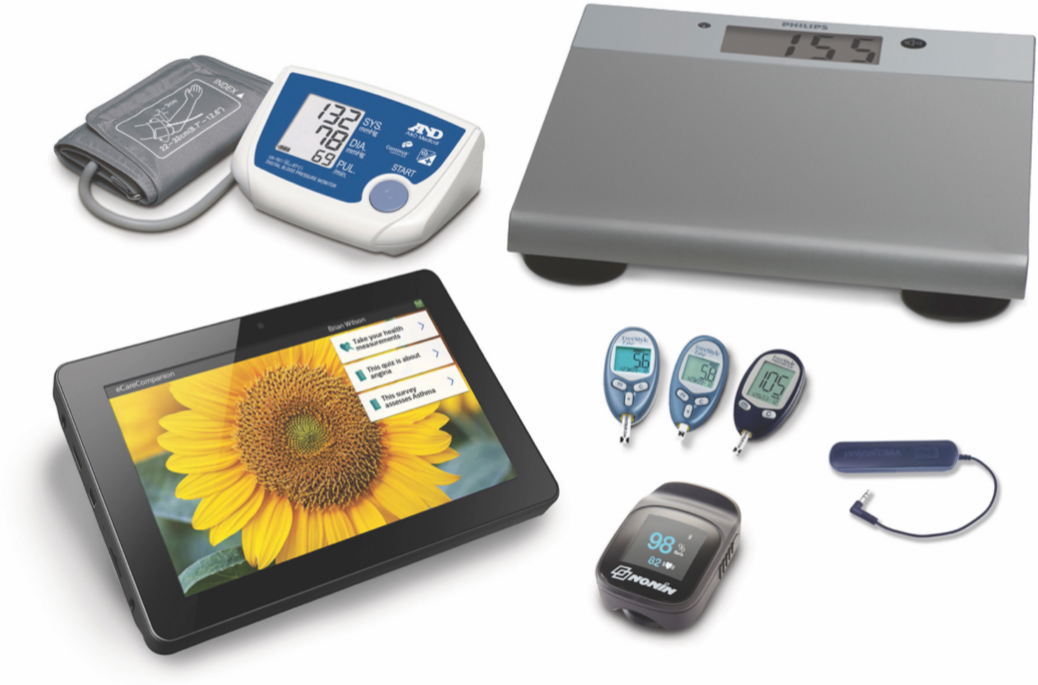
The Philips electronic Transition to Ambulatory Care system, featuring tablet interface and Bluetooth-enabled vital signs monitors. Reproduced with permission from Philips (reprinted with copyright permission from the publisher).

Hospital-to-home remote monitoring and virtual care is supported by the Philips Electronic Transition to Ambulatory Care (eTrAC) Program as shown in Figure 2, a tablet-based solution that combines clinical software for remote patient management with Bluetooth-enabled, vital signs monitoring equipment to measure SpO_2_, blood pressure, weight, core temperature, and blood glucose [1,9,19]. The nurse interface for eTrAC is eCare Coordinator (eCC). Through eCC secure video visits, nurses review patients’ vital signs and weight and conduct remote postoperative assessments daily. These standardized, daily assessments are designed to detect early signs of postoperative complications that may require medical intervention and to address patient concerns during recovery (eg, unrelieved pain in the moderate-to-severe range). Customizable daily patient symptom surveys (ie, general health, wound care, nutrition, medication reconciliation, sleep, functional status, and depression) are also collected via eTrAC [1,9,19]. Together, patient vital signs and survey responses factor into a weighted algorithm, which generates a daily triage score to prioritize nursing assessment and facilitate timely escalation of care to the most responsible physician member of the surgical team [1,9,19].

### Design

Our approach to Guardian and eTrAC system usability testing was guided by Wiklund et al’s *Usability Testing of Medical Devices* [20] and conducted with an *out-of-the-box* orientation. Out-of-the-box usability testing involves observing participants interacting with ready-for-use medical devices to perform required tasks, according to laid out instructions, in simulated real-world use case scenarios. As such, our intent was to conduct formative user tests, that is, tests focused on refining our approach to nurse and patient training, as well as refining system workflows with participant input [20].

### Setting and Recruitment

Participants included surgical ward nurses and patients recovering from cardiac or major vascular surgery. This study was conducted at two hospital sites, one in Ontario, Canada, and one in Liverpool, the United Kingdom. Nurse participants were recruited through brief presentations at staff meetings and nursing rounds, as well as through emails sent by ward managers. Nurses were invited to participate in either Guardian or eTrAC user testing, but not both, to avoid possible confounding influences of cross-system testing. Patients were invited to participate in eTrAC user testing only, given that Philips’ Guardian system does not involve active workflows for patients wearing the wireless sensors. Included patients were ambulatory, recovering from a cardiac or major vascular surgery, and were able to read, speak, and understand English. Patients who exhibited signs of postoperative delirium (via confusion assessment method) were excluded. The research personnel identified and approached eligible nurses and patients to participate in the study, obtained informed consent, collected baseline demographic information, and scheduled user testing sessions.

To account for site differences, we aimed to recruit a minimum of 6 participants per site for each system-related user test, for a total of 12 nurses for Guardian testing, 12 nurses for eTrAC testing, and 12 patients for eTrAC testing. Our total sample size of 36 participants was informed by Wiklund et al’s guidance [20] that five to six user test sessions per device are typically sufficient to identify usability issues in the context of a simulated out-of-the-box user testing.

The Hamilton Integrated Research Ethics Board approved the study (project reference number 2332). For the UK arm of the study, Coventry University Ethics Committee granted the ethical approval (project reference ID P50671). Research Governance approval was also granted by Dr Jay Wright, Liverpool Heart and Chest Hospital National Health Service Foundation Trust Research Committee as a Chairman’s action.

### Study Procedures

**Figure 3:**
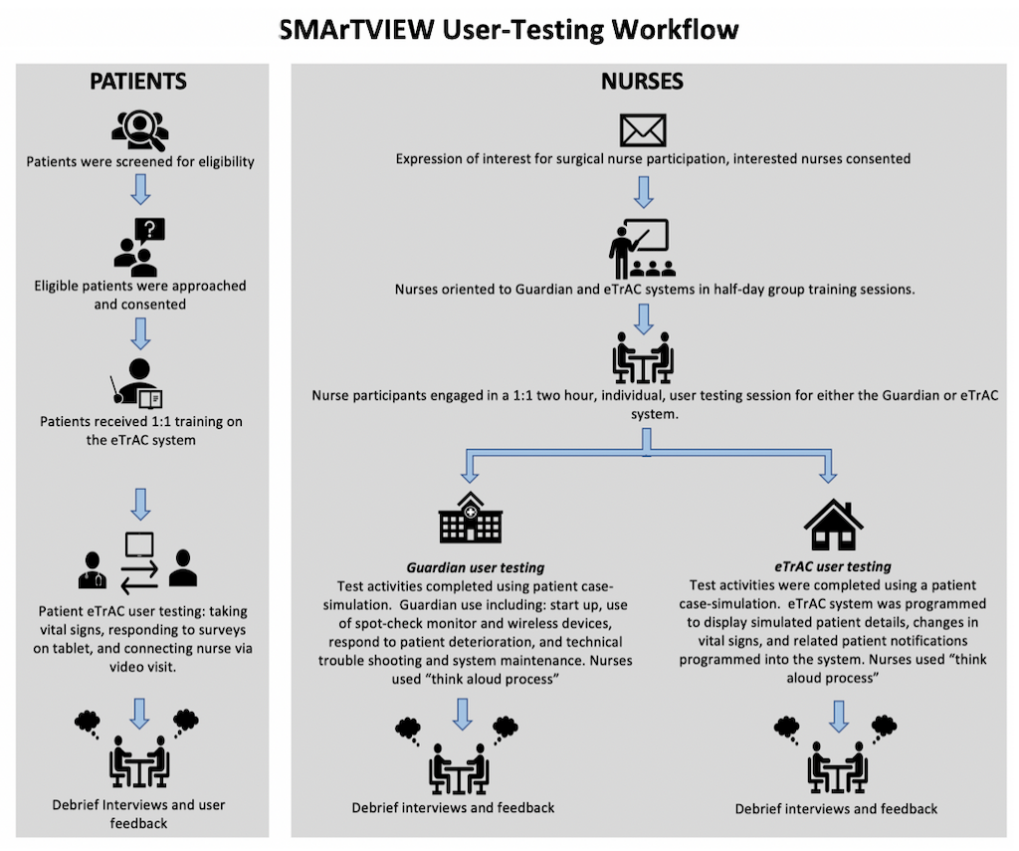
Nurse and patient user testing workflow diagram.

Usability testing of Guardian and eTrAC systems was conducted in two stages: hands-on systems training (Stage 1), followed by individual user testing (Stage 2; Figure 3). Individual user testing occurred as soon as possible after hands-on system training within 24 hours.

#### Hands-On Systems Training

##### Nurses

Nurse participants were oriented to Guardian and eTrAC systems during half-day group training sessions. These sessions included a combination of didactic and hands-on learning activities to contextualize and apply content. Topics included nurse and patient onboarding, system navigation, device management, monitoring functions, and technical troubleshooting and related communication. At the end of each session, nurse participants received system user guides for their review and ongoing reference. Nurse participants were asked to review these guides in preparation for their individual usability testing sessions.

##### Patients

Participating patients received individualized, 1-hour training sessions on the eTrAC system, also featuring didactic and hands-on learning. These sessions focused on daily use of the tablet and vital signs equipment and communicating with a nurse remotely via a secure video link. The training took place at a time convenient to the patient that did not interfere with their routine care. An eTrAC user guide was also provided.

#### Individual User Testing

##### Nurses

Following the systems training sessions, nurse participants took part in a 2-hour, individual, usability-testing session for either the Guardian or eTrAC system. The test activities were completed within the context of a simulation featuring one of two patient-cases, depending on nursing area of specialty—cardiac surgery (coronary artery bypass graft) or major vascular surgery (femoropopliteal bypass). Patient cases spanned postoperative days 1 through 4 and featured vital signs–related deteriorations that were programmed into the system remotely; the actors’ true vital signs were concealed. These cases were created based on real patient data by staff surgeons, residents, and nurses with minimum 5 years’ experience in managing patient deterioration at our study sites. The case scenarios were written in collaboration with the McMaster Standardized Patient Program, Centre for Simulation-Based Learning. A standard template was used to clarify required nurse end-user tasks, the setup of the simulation, and details of the patient cases including surgical details, past medical history, social and family history, and patient-related thoughts, feelings, and concerns that are common in the immediate postcardiac and postvascular surgery context. To provide a high degree of realism, 2 standardized patient actors were trained to portray each case; these actors were placed in an extra bed on the surgical unit at each site, in a separate area designated for the user testing [20].

###### Guardian User Testing

Following an introduction and review of the system, nurse participants completed the test activities, which represent core nursing competencies for Guardian use including start up, use of the MP5 spot-check monitor and wireless devices, ongoing monitoring and responding to patient deterioration, and technical trouble shooting and system maintenance.

The research assistant moderated the simulation by introducing the background information on the patient and providing instructions and cues to indicate advances in case timelines (eg, “It is now postop day 3 and you return to the unit and reassess your patient.”). The actors were trained to interact with the nurse participants to add realistic distractions and stressors similar to real-world working conditions on the surgical unit.

###### eTrAC User Testing

The eTrAC user testing followed procedures similar to the Guardian user testing with hospital-to-home patient cases. The eTrAC system was programmed to display simulated patient details, changes in vital signs, and related patient notifications programmed into the system. Patient cases (cardiac and vascular surgery) spanned postdischarge days 1 through 5 and featured vital signs–related deteriorations (eg, high temperature), indicative of common postoperative adverse events (eg, infection) while recovering at home. The research assistant prompted participants with details of these cases as they unfolded to indicate the passage of time and recovery-related circumstances for the patient at home.

##### Patients

The patient eTrAC user testing focused on completing system tasks required for using the eTrAC system at home, after hospital discharge. The research assistant asked participants to imagine they were now home and undertake the required daily patient-user test activities including using the vital signs equipment, responding to surveys on the tablet, and connecting with a nurse through a secure video visit. The eTrAC system was programmed to display simulated vital signs in demonstration mode. The portable vital signs monitors used by the participants did not display their actual vital signs.

#### Think Aloud Process

Across all user testing sessions, the research assistant asked participants to *think aloud* as they worked through required tasks [20]. Probing questions were asked to solicit participant reaction to each system and scenario, depending on participants’ apparent level of ease or difficulty as they worked through required tasks (eg, “How are you feeling at this point?”). While thinking aloud, nurse participants were asked to share any critical thinking they were engaged in as they responded to simulated patient deterioration, including problem solving and making decisions about escalating patient care to a physician team member as needed. Patient participants were asked to offer their reflections on their user experience. The research assistant did not respond to participant comments during the *think aloud* process [20].

### Outcome Data Collection

#### User Performance

The research assistant used an observation rubric to evaluate participants’ performance of test activities, according to the following designations: *completed independently*, *completed with difficulty or need for additional prompting*, or *not completed*. Additional observations recorded while participants engaged in the *think aloud* process included task completion time, distraction points, system navigation problems, and any areas of frustration or confusion about workflow when using the systems [20]. A second, silent observer recorded observational field notes to corroborate the research assistant’s observations. A mounted video camera was also used to record the simulations for verifying user performance and any discrepancies noted between participants’ subjective remarks and the documented observations [20].

#### User Acceptance

##### Perceived User Satisfaction

Participants were asked to rate their perceived satisfaction with the Guardian or eTrAC system user experience in the context of the workflow training we provided. To solicit this rating, we used the 11-point, single-item Net Promoter Scale (NPS) [21], which asks, “How likely is it that you would recommend the system to a friend or colleague”? We asked participants to respond to this question, while reflecting on the ease of system use during user testing. The NPS includes a score from 0 (*not at all likely*) to 10 (*very likely*). On the basis of response, individuals are classified as potential system *promoters* (response score: 9 or 10), *passives* (response score: 7 or 8), or *detractors* (response score: 0 to 6). A total NPS score, ranging from −100 to +100, can be calculated for each system tested by subtracting the percentage of those classified as *detractors* from the percentage of those classified *promoters*; scores above 0 indicate overall system-related satisfaction [21]. The NPS is widely used for system and health services tests [21-24], with moderate to strong correlation with other measures of patient and user satisfaction [25].

##### Debrief Interviews

Immediately following each user testing session, the research assistant conducted a 60-min semistructured debrief interview with participants to further identify root causes of any observed difficulties during the simulation, discuss required tasks that may have been missed, and solicit the participants’ overall impressions of their experience using the systems [20].

### Data Management and Analyses

Descriptive statistics were used to summarize participants’ demographic characteristics and user performance and acceptance of RAM systems and workflows. All qualitative data, including documented observations and audio recordings of the debrief interviews, were transcribed verbatim. Qualitative analysis was conducted employing inductive, thematic content analysis methods [26], with NVivo 10.0 software (QRS International). Data were coded based on the frequency, extensiveness, and specificity of participants’ comments as they related to the usability of Guardian and eTrAC systems [26]. These codes were altered and refined through a recursive process from the data to analyst-generated categorical and conceptual definitions. Revisions to the codebook reflected emerging themes. Constant comparative methods were used to examine individual participants’ responses in relation to responses from their respective user groups. New codes occurred less frequently as more transcripts were analyzed, and thematic saturation occurred for each user group when no new codes were generated. Rigor was maintained by completing a reflexive journal and audit trail [26].

## Results

### Demographics

#### Nurses

A total of 26 nurses from Canada (CAN group, n=15) and the United Kingdom (UK group, n=11) participated in the Guardian or eTrAC user testing. The majority of nurses were white females, possessing a bachelor’s degree in nursing, and employed full time. On average, these nurses had been practicing nursing for over 16 years, in cardiac and vascular surgery and other acute care settings as shown in Table 1.

**Table 1:** Nurse participant characteristics (n=26).

Nurse characteristics		Values
**Sex, n (%)**		
	Male	4 (15)
	Female	22 (85)
**Ethnicity, n (%)**		
	White	21 (81)
	African decent	4 (15)
	Asian	1 (4)
**Education, n (%)**		
	Professional degree	6 (23)
	Bachelor’s degree	11 (42)
	Masters’ degree	7 (27)
	Diploma	2 (8)
**Employment status, n (%)**		
	Full-time	19 (73)
	Part-time	7 (28)
Number of practicing years, mean (SD)		16.5 (12)

#### Patients

A total of 11 patients (CAN: n=6; UK: n=5), participated in the eTrAC user testing across both study sites. The majority of patients were male, either married or widowed, and retired. All patients were over 65 years of age; the majority had undergone coronary artery bypass graft or valve replacement surgery as shown in Table 2).

**Table 2:** Electronic Transition to Ambulatory Care patient participant characteristics (N=11).

Characteristics		Values, n (%)
**Sex**		
	Male	7 (64)
	Female	4 (36)
**Ethnicity**		
	White	11 (100)
**Marital status**		
	Married	7 (64)
	Widowed	3 (27)
	Divorced or separated	1 (9)
**Education**		
	Some high school—no diploma	5 (46)
	High school diploma	3 (27)
	Trade, technical, vocational training	1 (9)
	Professional degree	2 (18)
**Employment status**		
	Full-time	2 (18)
	Part-time	3 (27)
	Retired	6 (55)
**Procedure**		
	Coronary artery bypass graft	6 (55)
	Abdominal aortic aneurysm repair	1 (9)
	Heart valve replacement	4 (36)

### User Performance

User performance, expressed as percentage of participants observed by task category, that is, *completed*, *completed with difficulty or additional prompting*, and *not completed*, and median task completion times are presented in Table 3.

**Table 3:** User performance.

User (N) and task		Completed, n (%)	Completed with difficulty or additional prompting, n (%)	Not completed, n (%)	Task completion time (mm:ss), median (IQR)
**Guardian nurse: 8 CAN^a^; 6 UK^b^ (N=14)**					
	Nurse pairing the patient to the monitor	11 (79)	3 (21)	0 (0)	00:10 (00:05-00:11)
	Assign wireless devices to the patient	9 (64)	5 (36)	0 (0)	04:30 (03:10-06:16)
	Complete full set of vital signs	14 (100)	0 (0)	0 (0)	01:04 (00:40-01:47)
	Validate EWS^c^	13 (93)	0 (0)	1 (7)	00:03 (00:02-00:05)
	Review and manage the patient’s vital sign trends	8 (58)	6 (42)	0 (0)	00:26 (00:17-00:47)
	Wireless device management	12 (87)	2 (13)	0 (0)	02:08 (01:10-03:17)
	Infection control procedures	14 (100)	0 (0)	0 (0)	01:03 (00:30-01:50)
**eTrAC^d^** **patient: 6 CAN; 5 UK (N=11)**					
	Turn on device	9 (82)	1 (9)	1 (9)	00:08 (00:05-00:15)
	Take vital signs (BP^e^, SpO_2_^f^, weight, HR^g^, temperature)	8 (73)	3 (27)	0 (0)	01:01 (00:45-01:14)
	View scheduled appointment in the calendar	11 (100)	0 (0)	0 (0)	00:22 (00:18-00:43)
	Engage in follow-up surveys	9 (82)	2 (18)	0 (0)	00:16(00:12-00:20)
	Interface with nurse	10 (91)	1 (9)	0 (0)	00:16 (00:06-00:39)
**eTrAC nurse: 7 CAN; 5 UK (N=12)**					
	Log in and enroll new patient	11 (92)	1 (8)	0 (0)	00:41 (00:31-01:18)
	Assign H2H^h^ protocol	6 (50)	5 (42)	1 (8)	01:55 (01:14-02:21)
	Assign BTE^i^ devices	0 (0)	11 (92)	1 (8)	03:35 (03:10-04:47)
	Review score and triage the patient	5 (42)	6 (50)	1 (8)	00:47 (00:28-01:07)
	Video call and patient wound photo	12 (100)	0 (0)	0 (0)	02:35 (01:57-03:49)
	Appropriate escalation of care	12 (100)	0 (0)	0 (0)	Verbal response (not timed)
	Add clinical notes	12 (100)	0 (0)	0 (0)	01:12 (00:46-01:34)

^a^CAN: Canada.

^b^UK: United Kingdom.

^c^EWS: early warning score.

^d^eTrAC: electronic transition to ambulatory care.

^e^BP: blood pressure.

^f^SpO_2_: blood oxygen saturation.

^g^HR: heart rate.

^h^H2H: hospital-to-home.

^i^BTE: Bluetooth-enabled.

#### Guardian System

The majority of nurse participants were able to complete most required tasks independently, demonstrating comprehension and retention of required Guardian system workflows, for example, prompting the system to complete a set of *on demand* vital signs. Tasks, which required additional prompting by the facilitator, for some, were related to the use of system features that enable continuous patient biometric data transmission [9,18] and active clinical management of this information (eg, assigning wireless devices to the patient and displaying continuous patient vital signs trends on the central monitor).

#### eTrAC System

Nurse users demonstrated confidence with eTrAC workflows related to onboarding patients onto the system, as well as direct patient interaction and remote patient management, including assessment, documentation of independent nursing actions, and escalation of care to the most responsible physician in the patient case scenario. These users were less confident in working with system protocols for remote wireless patient vital signs transmission, such as assigning the Bluetooth-enabled vital signs devices to the patient for home use and assigning the appropriate hospital-to-home monitoring regimen based on surgical procedure.

The majority of patient users demonstrated ease and independence with all required eTrAC tasks. Similar to nurses, some required additional prompting to work with the Bluetooth devices to take their vital signs and navigate aspects of the system interface related to remote self-monitoring (eg, responding to symptom survey).

### User Acceptance

#### User Satisfaction—Net Promoter Scale score

Individual and mean NPS ratings, by user group, are presented in Table 4. Mean scores indicate a high degree of likelihood that each user group would recommend the Guardian and eTrAC systems to others, based on their training and user test experience. Overall NPS scores, by user group (nurses and patients), also indicate overall user satisfaction with each system.

**Table 4:** User satisfaction—Net Promoter Scale score.

User (N) and group		Raw scores (range 0-10)	Value, mean (SD)	NPS^a^ score (% of promoter−% of detractors)
**Guardian nurses: 8 CAN^b^; 6 UK^c^ (N=14)**			8.8 (0.89)^d^	64
	Nurse 1	8		
	Nurse 2	8		
	Nurse 3	8		
	Nurse 4	8		
	Nurse 5	7		
	Nurse 6	9		
	Nurse 7	10		
	Nurse 8	10		
	Nurse 9	10		
	Nurse 10	9		
	Nurse 11	9		
	Nurse 12	9		
	Nurse 13	9		
	Nurse 14	9		
**eTrAC^e^ nurses: 7 CAN; 5 UK (N=12)**			7.7 (1.4)^d^	25
	Nurse 1	4		
	Nurse 2	7		
	Nurse 3	8		
	Nurse 4	7		
	Nurse 5	8		
	Nurse 6	8		
	Nurse 7	8		
	Nurse 8	8		
	Nurse 9	9		
	Nurse 10	9		
	Nurse 11	9		
	Nurse 12	9		
**eTrAC patients: 6 CAN; 5 UK (N=11)**			9.2 (0.75)	82
	Patient 1	8		
	Patient 2	8		
	Patient 3	9		
	Patient 4	10		
	Patient 5	10		
	Patient 6	10		
	Patient 7	9		
	Patient 8	9		
	Patient 9	9		
	Patient 10	9		
	Patient 11	10		

^a^NPS: Net Promoter Scale.

^b^CAN: Canada.

^c^UK: United Kingdom.

^d^This is the average score.

^e^eTrAC: electronic transition to ambulatory care.

#### Debrief Interviews

Posttest debrief interviews provided opportunities for users to reflect on their own performance and how they felt during the test simulations, any areas of difficulty that they had, and what (if any) improvements to system workflow training could be made. The participant’s overall accounts of their user experience—positive or negative—were also solicited.

##### Nurses

Key themes that emerged from the interviews of nurses engaged in the Guardian user testing related to system *ease of use*, vital signs *trend monitoring*, and *wireless device management*.

In terms of *ease of use*, most users spoke of the simplicity of the Guardian interface and feeling confident about navigating the system after a short while. Participants also commented on their perception of Guardian as an aide to day-to-day nursing work on busy surgical wards:

It does the job...more frequently than a nurse can. We are only one nurse taking care of 4 to 5 patients, so if our monitor can do continuous monitoring and alert us when our patient is [deteriorating], that’s unbelievable.

UK Nurse, participant 002-009

Some nurses cited unfamiliarity with more advanced aspects of the system as a barrier to engaging in vital signs *trend monitoring* during the simulations. However, no participants expressed that changes would be needed to the system or related workflow training. Rather, they emphasized the importance of more opportunities to practice and get comfortable with software features that enable management and visualization of continuous patient vital signs data. Others commented that having the ability to examine vital signs trends remotely would be invaluable to patient management:

I thought that was the coolest thing. You can monitor your patient from [your handled device], the desktop central station, or from the monitor, at the bedside. So you don’t have to be at the bedside all the time to know [patient status].

CAN Nurse, participant 001-005

Reflections on *wireless device management*, were also indicative of excitement about the potential for remote monitoring to improve patient safety and create efficiency in nursing work through automation of time-consuming processes, such as manual data entry:

I think it’s going to be really safe. If I can constantly know that they’re [patients] going to be okay if I leave them and do other things and it’s just very quick and easy.

UK Nurse, participant 002-009

I think it’s awesome that it is barcodes and scanning and no data entry. I think half our shifts are wasted with data entry—we don’t need any more of that!

CAN Nurse, participant 001-003

Nurse participants who were debriefed following the eTrAC user testing reflected on this system as an enabler of their *clinical nursing skills*, as well as an opportunity and need to learn new *technical skills*. Nurses found that the hospital-to-home simulation allowed them to use their critical thinking skills by assessing the patient directly through the eTrAC video feature, and by reviewing vital signs and patient survey results before they conducted their assessments. Many felt that the clinical interface was well designed and simple to navigate, allowing for a complete picture of the patient and timely decisions about clinical action:

It’s quite cool to see all your patients and the alerts. Because, you know, you could have twenty patients on there, and they’re all fine, making a good recovery. I like the way the alerts and the scores are visible and you can act on it straight away.

UK Nurse, participant 002-014

Most participants commented that they needed additional prompting to assign hospital-to-home protocols and to assign the Bluetooth-enabled vital signs devices to the patient and tablet. Lack of familiarity and confidence with the technical aspects of the eTrAC interface were discussed as key challenges to completing these tasks. When asked, all participants said they would need and would welcome the opportunity to develop these technical skills further, and that with additional support, they could see themselves becoming proficient in these aspects of eTrAC use.

##### Patients

Patient debrief interviews revealed an overall positive experience with the eTrAC user testing, with *ease of system use* and *recovery progress* as key themes that emerged. When asked for feedback on navigating the patient tablet, participants commented that it was both pleasing aesthetically and uncomplicated operationally. Specifically, patients indicated that the interface was clear and easy to use, and that the font was legible. Despite half of these patients noting that they had initial anxiety about using the technology, most remarked feeling comfortable once they began user testing. As one participant commented:

At first I thought I couldn’t do it but again, like I said, it is very straightforward. Very easy, you start at the top and just finish the temperature or the height, or your weight, and you just follow the tablet.

CAN patient, participant 001-009

All participants remarked that they felt the system would be invaluable for helping them through recovery. There was a high degree of enthusiasm about connecting with a nurse daily and being monitored, as it would offer a sense of security after hospital discharge:

I think that’s the best thing of all... there’s somebody at the end of that—just like telephone line, there’s somebody at the end of that you can talk to and you can see them... that’s really good.

CAN patient, participant 001-017

Participants also commented on the value of engaging in eTrAC patient surveys to give a monitoring nurse more information and ensure that they are on track with recovery:

I would give it [survey feature] a 10 plus, plus. I think it is just comforting to know that you are on the right track and can tell the nurse what’s happening.

CAN patient, participant 001-021

In summary, participant debrief interviews indicated a high degree of acceptance among users. Nurses expressed the importance and potential of remote monitoring and virtual hospital-to-home care as means to improve efficiency of clinical workflows, enhance patient safety, and facilitate timely clinical action. Patient users spoke to the security that these systems can offer through daily connection with a nurse while recovering from surgery. Both user groups stressed the need for additional opportunities to practice in order to become comfortable and proficient in the use of the Guardian and eTrAC systems, with respect, in particular, to mastering more technical aspects related to enabling remote connectivity and assigning and engaging in monitoring protocols.

## Discussion

### Principal Findings

This study addressed user performance and acceptance of Philips’ Guardian and eTrAC systems designed to support in-hospital RAM and virtual care from hospital-to-home, respectively [9,18,19]. The planned use of these systems in combination succession, within a trial intervention, is a unique approach to studying the effects of enhanced patient surveillance and remote recovery support following cardiac and major vascular surgery [1,9]. *Out-of-the-box* user testing [20] uncovered overall strong user performance by both nurses and patients for the majority of required user tasks. Testing also uncovered important areas where our approach to systems training and implementation needed to be strengthened to better support users. With respect to Guardian, a number of participants required additional support from the facilitator to complete more advanced system-related tasks that would enable acquisition and transmission of continuous patient vital signs data monitoring, such as pairing of the wireless sensors to the MP5 spot-check monitor. Results of eTrAC testing were similar, suggesting that nurse users were less confident after training when it came to pairing Bluetooth-enabled monitoring devices to the patient tablet, as well as assigning specific remote monitoring protocols (related to surgical type).

These user performance results were used to enhance our approach to systems training during SMArTVIEW trial start up at participating hospital sites. Initial systems in-services for nursing staff were followed by individual facilitated practice sessions both in classroom settings and on the surgical wards. These applied learning opportunities allowed for the development of ward nurses’ required technical skills to become proficient in the use of Guardian, while they transitioned from case-based learning to live systems use. Some nurse participants also became designated Guardian *champions* at SMArTVIEW trial sites, acting as resources for other ward nurses to support ongoing learning and technical trouble shooting.

Given that the use of the Philips eTrAC hospital-to-home system [19] represents a skill set unique from standard ward nursing, it was decided that deployment of the system, at each study site, would be preserved for a designated subteam of nurses (seconded from ward duties), who focus on hospital-to-home virtual care. These specialized nurses, referred to as SMArTVIEW nurses, take responsibility for onboarding study patients to the eTrAC system and monitoring them for the first 30 days at home following hospital discharge [9]. These nurses provide daytime hospital-to-home service for patients allocated to the intervention arm of the SMArTVIEW trial, 7 days per week, from a designated space on the surgical ward. In this role, they also assist ward nurses with Guardian implementation at the beginning of each shift to reinforce systems training and optimize adoption of RAM workflows.

### Comparison With Prior Work

This study demonstrated a high degree of acceptance in terms of user satisfaction and communication of a positive learning experience, highlighting the value of providing risk-free opportunities to learn RAM and virtual care technologies, before implementation, to ease end-user apprehensions and achieve buy-in. A few other studies have examined specifically user experience in the context of RAM and virtual care technology planning or pilot testing in surgical settings. In their recent multimethod study to examine nurse and physician perceptions of a planned introduction of continuous RAM on general hospital wards, Prgomet et al [14] found that hospital staff first expressed apprehension and beliefs about RAM technologies that would likely counter successful implementation. Nurses were concerned about the potential for staff overreliance on RAM technology and, hence, reduced bedside patient interaction and examination. Physicians were concerned about inappropriate care escalations based on false-positive RAM notifications and subsequent desensitization to alerts. Both groups expressed similar concerns over the potential for alarm fatigue [14].

As was the case in our study, however, the opportunity to trial the monitoring devices and engage in dialogue about their impact on clinical workflows and patient care gave rise to perceptions that focused training, featuring educational opportunities to address pre-existing attitudes and beliefs about the incorporation of RAM technologies into clinical practice, would be an important prerequisite to successful implementation [14]. Despite initial concerns, there was also acknowledgment by nurses of the potential for RAM devices to enhance early detection of patient deterioration and provide supporting evidence when communicating concerns about patient status to physician colleagues [14].

In a prospective study (N=443), McElroy et al [27] incorporated a digital health kit—featuring a patient tablet and Bluetooth-enabled vital signs monitors (with software enabling abnormal vital signs to trigger automated alerts to clinicians)—into a 30-day readmission reduction program following cardiac surgery. Posttest user satisfaction survey results were positive among patient and nurse respondents. Similar to this study, the high degree of user satisfaction observed was attributed to the simplicity of the digital kit system, including easy-to-use tablet software and system configuration that allowed for easy patient remote connection to a nurse by video [27].

The VItal siGns monitoring with continuous puLse oximetry And wireless cliNiCal notification aftEr surgery study investigators [28] conducted an evaluation report to uncover nurse and patient user perspectives on RAM implementation challenges during a recent RCT (N=2049) of continuous vital signs monitoring with alerts to nursing staff on the incidence of respiratory resuscitations, code blues, and intensive care unit transfers in patients undergoing noncardiac surgery. A key challenge to implementation was nurse adherence to required clinical workflow changes to accommodate RAM. Nearly 23% of records used to track nursing compliance with the RAM intervention were missing, indicating compliance issues despite ongoing ward training [28]. Restriction to ambulation imposed by the continuous pulse oximetry cable and sensor-related discomfort were noted as common reasons for patient withdrawal (10.68%) from the RAM intervention. A key recommendation of the evaluation report was that pre-emptive user testing would be important for future RAM studies and clinical applications to achieve stakeholder buy-in and co-design and refinement of clinical workflows, as well as to establish RAM champions during implementation [28]. Other studies soliciting nurse perspectives following RAM pilots report similar recommendations [15,29] and the need for constant attention to change management.

### Limitations

Potential limitations of this study include our homogenous participant sample, as well as our approach to measuring task completion times and level of interaction with participants during the *think aloud* process [20]. Our user testing procedures relied on a convenience sample of nurses and patients who were in-hospital and agreed to participate at the time of testing. Those enrolled were white individuals who spoke English. Although user testing studies are not designed for generalization to broad populations per se, our results speak to user acceptance and performance in a limited subpopulation of end users. It should also be noted that of the 11 patients enrolled, just 1 eligible patient had undergone vascular surgery at the time of study enrollment—the remainder of the patient sample was cardiac surgery patients. Nonetheless, the equipment and workflows tested were designed to be identical for both patient groups.

As is common during formative types of user tests that are intended to reveal shortcomings in systems training or workflows [20], some of our recorded individual task completion times may have been distorted. Task time distortions occur when test participants pause to reflect on completion of their user tasks while thinking aloud [20]. An approach to remedying task time distortion is to require participants to remain silent during user testing. However, this approach is more amendable to summative user tests [20], which place greater importance on measurement precision over participant narrative about their user experience. In this study, we favored concurrent participant observation and moderation over achieving recorded task time precision; this approach allowed us to identify clear gaps in systems training and aspects of workflow implementation that needed refining before launch of the SMArTVIEW trial.

We also undertook a flexible approach to participant moderation during the *think aloud* process. During test simulations, the facilitator prompted participants with key words or instructions if they were clearly struggling with certain required tasks. This strategy allowed us to include a *completed with difficulty or need for additional prompting* category in our user performance rubric. Although less conventional than traditional formative user testing [20], we again placed high value on the solicitation of user narrative. As with task completion times, we were more concerned with participant involvement in as many aspects of Guardian and eTrAC workflows as possible (and related reflection) than with the generation of conventional user performance metrics.

### Conclusions

The inadequacy of current systems for postsurgical patient monitoring in hospital and at home is a major factor contributing to postoperative complications, death, and unplanned hospital readmissions [6-10]. Although RAM and virtual care technologies have high potential for improving postoperative patient outcomes, the science of implementing and evaluating these technologies is complex and still at an early stage. This formative *out-of-the-box* user testing study indicated a high degree of user acceptance of Philips’ Guardian and eTrAC systems among nurses and patients. Key insights were also provided that informed refinement of clinical workflow training and systems implementation, including clear division of responsibilities, before launch of the international SMArTVIEW trial. This trial, underway, is designed to examine the effectiveness of postoperative RAM and virtual hospital-to-home recovery support on health system and patient-related outcomes following cardiac and major vascular surgery. Practical implementation issues will also be explored, including the need for specialized training of subteams of nurses to deploy RAM and virtual hospital-to-home recovery support at trial sites.

## Abbreviations

eCC: eCare Coordinator
eTrAC: electronic transition to ambulatory care
NPS: Net Promoter Scale
RAM: remote automated monitoring
RCT: randomized controlled trial
SpO_2_: blood oxygen saturation
THE SMArTVIEW, CoVeRed: TecHnology-Enabled remote monitoring and Self-MAnagemenT—VIsion for patient EmpoWerment following Cardiac and major VasculaR surgery
